# Phenethyl Isothiocyanate and Cisplatin Co-Encapsulated in a Liposomal Nanoparticle for Treatment of Non-Small Cell Lung Cancer

**DOI:** 10.3390/molecules24040801

**Published:** 2019-02-22

**Authors:** Mengwei Sun, Yi Shi, Utkarsh J. Dang, Anthony J. Di Pasqua

**Affiliations:** 1 Department of Pharmaceutical Sciences, School of Pharmacy and Pharmaceutical Sciences, Binghamton University; Johnson City, NY 13790, USA; msun22@binghamton.edu (M.S.); yishi@binghamton.edu (Y.S.); 2 Department of Health Outcomes and Administrative Sciences, School of Pharmacy and Pharmaceutical Sciences, Binghamton University; Johnson City, NY 13790, USA; udang@binghamton.edu

**Keywords:** NSCLC, liposomes, cisplatin, isothiocyanate, chemotherapy, toxicity

## Abstract

Lung cancer is the leading cause of cancer-related death in the Unites States, and approximately 85% of all lung cancers are classified as non-small cell lung cancer (NSCLC), which is extremely difficult to treat and its survival rate is low. After decades of clinical trials, the most effective treatments are still those that implement the first-generation platinum anticancer agent cisplatin (CDDP) in combination with other drugs. We previously demonstrated that the naturally-occurring compound phenethyl isothiocyanate (PEITC) can be used to sensitize NSCLC cells to CDDP. Furthermore, co-encapsulation of PEITC and CDDP in liposomes enhances their toxicity toward NSCLC cells. We here optimize liposomal-PEITC-CDDP, demonstrate the release of PEITC and CDDP from the nanoparticle, and show that liposomal-PEITC-CDDP is much more toxic toward both A549 and H596 human NSCLC cell lines than toward WI-38 and BEAS-2B human normal lung cell lines. Thus, we have prepared an efficacious therapy that has significantly higher toxicity toward cancer cell lines than normal cell lines.

## 1. Introduction

Lung cancer is the leading cause of cancer-related death in the United States [1]. Approximately 85% of all lung cancers are classified as non-small cell lung cancer (NSCLC), which is extremely difficult to treat and its survival rate is low [1]. After decades of clinical trials, the most effective treatments are still those that implement the first-generation platinum anticancer agent cisplatin (CDDP) in combination with other drugs [2]. Tubulin-binding agents, such as paclitaxel, are often used in combination with CDDP for treatment [3]. Microtubules, which are assembled from dimers of α-and β-tubulin, are critical for cell division, making tubulin proteins an important target in rapidly dividing NSCLC cells. Unfortunately, both CDDP and the agents used in combination with CDDP are toxic to the patient. Furthermore, tumors often gain resistance to CDDP. One of the major issues associated with CDDP is its nephrotoxicity, and when this is coupled with the neurotoxicity associated with the typically used tubulin binding agents, taxanes and vincas [3,4], the patient experiences significant side-effects. Thus, a treatment modality that aggressively decreases tumor volume, but has less toxicity to the patient has been sought. It has been demonstrated that the liposomal CDDP formulation LipoPlatin^TM^ is less toxic to patients than free CDDP is, but has a similar efficacy against NSCLC [5]. In a randomized clinical trial, the effectiveness of LipoPlatin^TM^ with the βx-tubulin binding agent paclitaxel was similar to that of CDDP with paclitaxel; however, much less toxicity to the patient was observed [6]. Not all tubulin binding agents are neurotoxic; indibulin, for example, was reported to have specificity for unmodified microtubules and, thus, no neurotoxicity in preclinical studies [7].

In 2010, we showed that naturally occurring isothiocyanates (ITCs) can be used to enhance the efficacy of CDDP [8]; ITCs are metabolites of glucosinolates which are contained in cruciferous vegetables such as watercress [9]. In the aforementioned study, structural variations among the naturally-occurring ITCs affected their ability to sensitize NSCLC cells to CDDP, and this correlated well with their ability to bind to and, ultimately, degrade β-tubulin in NSCLC; the ability of benzyl ITC (BITC) is about the same as that of phenethyl ITC (PEITC), both of which are much greater than that of sulforaphane (SFN) [10]. Thus, this mechanism may be important for said sensitization. This mechanism was further elucidated in a study demonstrating that allyl isothiocyanate enhances CDDP toxicity against lung and ovarian cancer cells [11].

In a separate report, a synthetic ITC derivative, ethyl 4-isothiocyantobutanoate, was shown to sensitize ovarian cancer cells to CDDP and, moreover, it allowed for CDDP to overcome CDDP-resistance in cell culture [12]. Then, it was shown that naturally-occurring ITCs sensitize cervical and breast cancers to CDDP, but not human normal mammary epithelial cells [13], and, in another study, the naturally-occurring ITC BITC sensitized leukemia cells to CDDP, but not normal human lymphocytes [14]. These results, combined with the observation that micromolar plasma concentrations of ITCs are safely maintained in humans [15], suggest that using ITCs with CDDP may be an efficacious treatment modality with fewer side effects than those currently in use.

In 2014, we reported on the preparation of a liposomal nanoparticle containing both PEITC and CDDP [16]. Studies had shown that drugs reformulated in liposomes have an increased circulation time in the bloodstream and, furthermore, increased accumulation in tumors, which is due to the enhanced permeability and retention (EPR) effect [17]. Liposomes containing the relatively hydrophilic CDDP and hydrophobic PEITC were prepared and characterized. For this preparation, 1,2-distearoyl-sn-glycero-3-phosphocholine (DSPC) was chosen. Liposomes composed of DSPC were previously shown to have greater drug retention over 48 h at 4 and 37 °C than those composed of phospholipids with lower phase transition temperature values [18]. These nanoparticles were uniform, with a size of approximately 140 nm, and had a zeta potential of approximately −65 mV, which indicates high stability of the formulation. The liposomal-PEITC-CDDP formulation was more toxic toward H596 cells than the combination co-administered as free drugs and liposomes containing only CDDP or PEITC.

We here expand on our previous work. Again, DSPC was used; however, the optimized liposomal nanoparticles have a 1:3 ratio of CDDP to PEITC, whereas the previously reported nanoparticles had a 1:2 ratio, so less CDDP can be administered. Careful characterization studies were performed and release profiles obtained for liposomal-CDDP, liposomal-PEITC and liposomes containing both CDDP and PEITC: liposomal-PEITC-CDDP. Then, toxicity was tested in two human NSCLC cell lines, A549 and H596, and compared to its toxicity in human normal lung cells, BEAS-2B and WI-38.

## 2. Results and Discussion

Blank and drug-encapsulated liposomes were prepared with the combination of DSPC:EPG in the ratio of 4:1. Various quantities of PEITC and/or CDDP were added to the liposomal formulation (Table 1). Percent loading and encapsulation efficiency (EE) of the two drugs increased with their concentrations, respectively (Table 1). The highest loading (1.35 ± 0.27%) and EE (83.9 ± 4.1%) for CDDP in liposomes (Lipo-CDDP) was achieved using 8.33 µmol of CDDP; the highest loading (3.66 ± 0.35%) and EE (37.0 ± 2.4%) for PEITC in liposomes (Lipo-PEITC) was achieved using 134 µmol of PEITC. In the PEITC and CDDP-loaded liposomal formulation (Lipo-PEITC-CDDP), 8.33 µmol of CDDP and 134 µmol of PEITC were used to obtain 1.37 ± 0.18% and 3.24 ± 0.47% loading of CDDP and PEITC, respectively.

**Figure 1:**
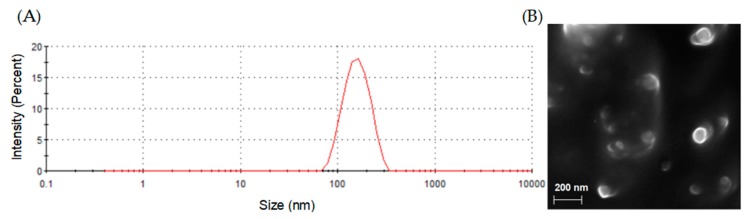
(**A**) Size distribution image of liposomes containing both phenethyl isothiocyanate (PEITC) and cisplatin (CDDP) (Lipo-PEITC-CDDP) and (**B**) SEM image of Lipo-PEITC-CDDP.

Particle size distributions and zeta potentials of blank and PEITC and/or CDDP loaded liposomes were characterized using a Zetasizer. As shown in Table 2, the average diameter of these liposomes ranged from 116.3 (blank liposomes) to 173.4 nm (Lipo-PEITC-CDDP). The average polydispersity indexes (PDI) of these nanoparticles indicate uniform particle size and good dispersion. The stability of the blank liposomes and drug-encapsulated liposomes was verified by measuring their zeta potentials, which ranged from −40 to −60 mV. It is shown in Figure 1A that Lipo-PEITC-CDDP has a narrow size distribution, which indicates uniformity. Field Emission Scanning Electron Microscope (FESEM) was used to characterize the morphology of Lipo-PEITC-CDDP. SEM observation confirmed that the Lipo-PEITC-CDDP exhibits spherical morphology, with an average particle size of approximately 150 nm.

**Figure 2:**
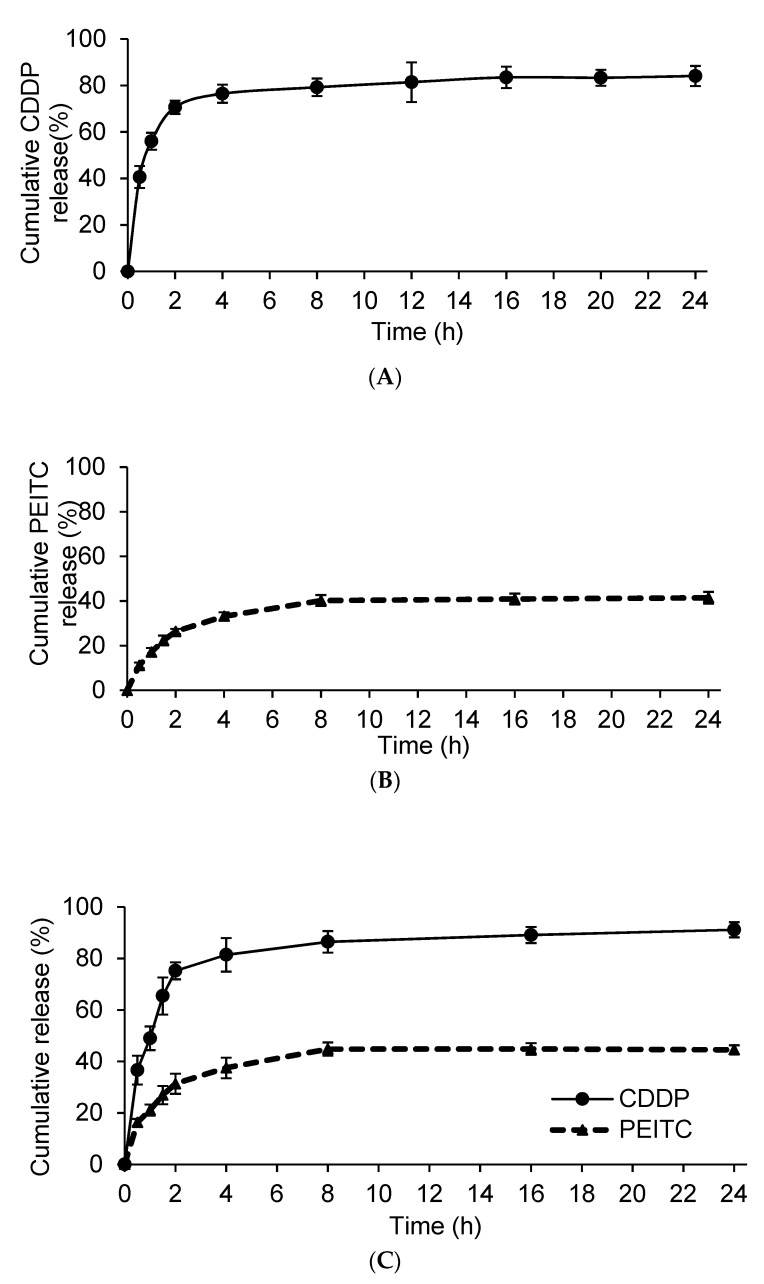
(**A**) In vitro cisplatin (CDDP) release profile from liposomes containing CDDP (Lipo-CDDP); (**B**) in vitro phenethyl isothiocyanate (PEITC) release profile from liposomes containing PEITC (Lipo-PEITC); and (**C**) in vitro CDDP and PEITC release profiles from liposomes containing both compounds (Lipo-PEITC-CDDP).

Drug release of nanoparticles is an important factor in determining the biological efficiency of drug delivery vehicles. Plots of percentage of CDDP and/or PEITC released from liposome formulations versus time are shown in Figure 2. The amount of CDDP released from Lipo-CDDP and Lipo-PEITC-CDDP over 24 h was measured using inductively-coupled plasma-mass spectrometry (ICP-MS). As is shown in Figure 2A and 2C, Lipo-CDDP and Lipo-PEITC-CDDP release 76.5 ± 3.9% and 75.2 ± 3.3% of their CDDP, respectively, in 2 h. Over 24 h, the amounts of CDDP released reached 86.1 ± 4.3% and 91.1 ± 2.9% for Lipo-CDDP and Lipo-PEITC-CDDP, respectively. A 1,2-benzenedithiol (BDT) assay was used to obtain the PEITC release profile of Lipo-PEITC and Lipo-PEITC-CDDP [19]. As is shown in Figure 2B, Lipo-PEITC showed a relatively sustained PEITC release, with 40.2 ± 2.5% PEITC released gradually from Lipo-PEITC in the first 8 h. The percent release remained almost the same up to 24 h. A similar PEITC release profile was observed in Lipo-PEITC-CDDP (Figure 2C) with 44.8 ± 2.6% of the PEITC released gradually within 8 h. The release rate of CDDP in Lipo-PEITC-CDDP was higher than that of PEITC.

**Figure 3:**
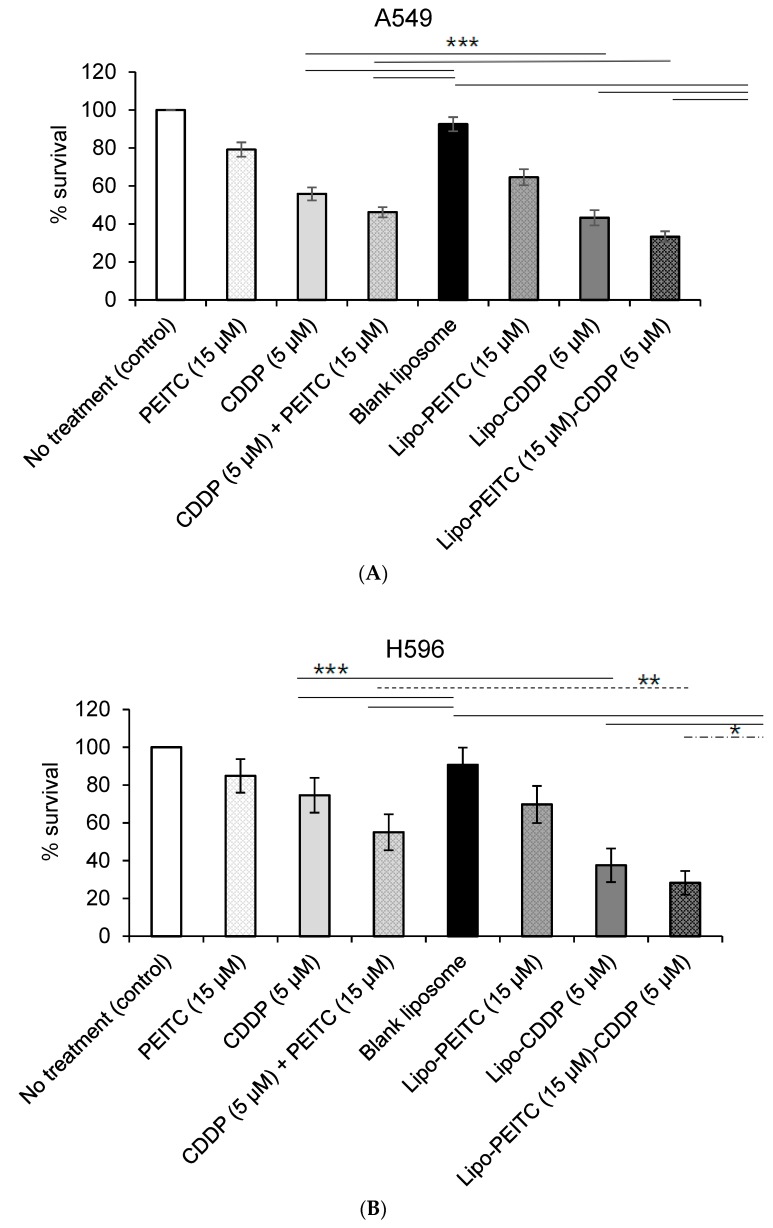
Percent survival of (**A**) human non-small cell lung cancer A549 and (**B**) H596 cells treated with free cisplatin (CDDP) and/or phenethyl isothiocyanate (PEITC), and liposome encapsulated compounds (solid line stands for ***p < 0.005, dash line stands for **p < 0.01 and long dash dot line stands for *p < 0.05).

**Figure 4:**
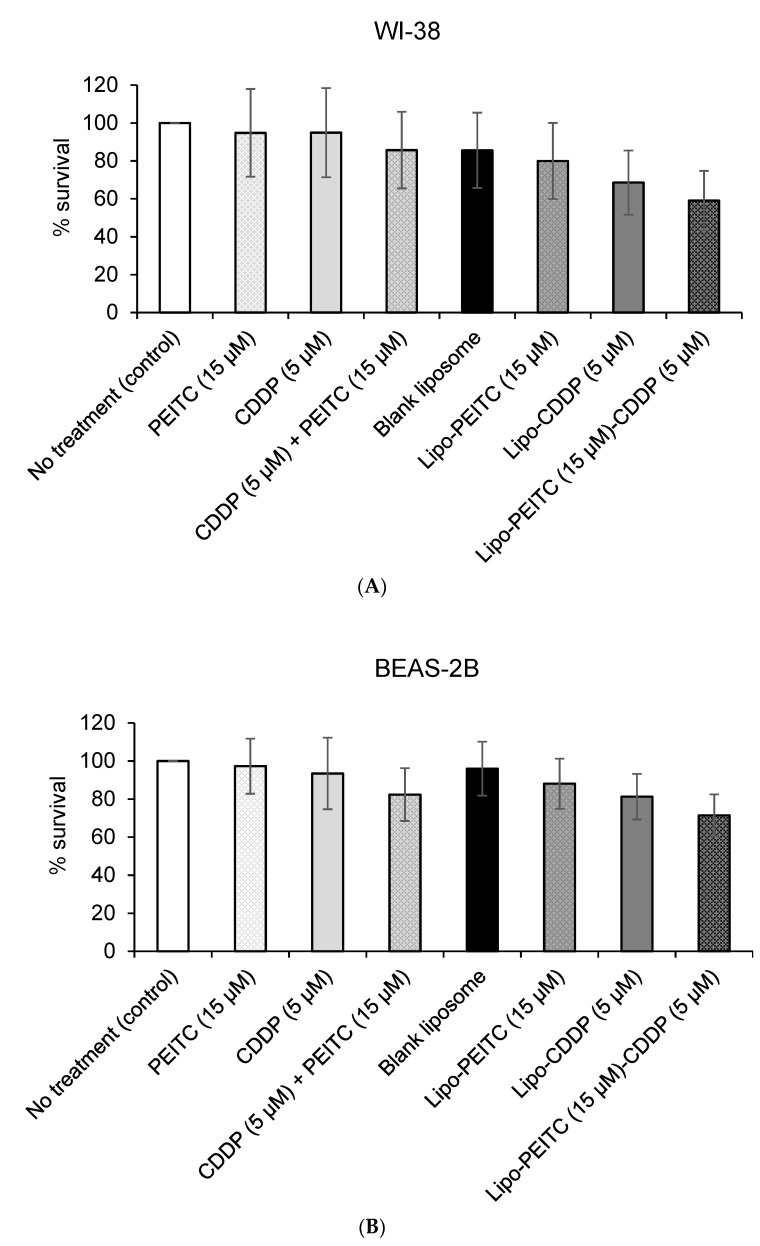
Percent survival of normal human lung cell lines (**A**) WI-38 (human diploid lung fibroblasts) and (**B**) BEAS-2B (human bronchial epithelial cells) treated with free cisplatin (CDDP) and phenethyl isothiocyanate (PEITC) and liposome-encapsulated compounds.

The MTS (3-(4,5-dimethylthiazol-2-yl)-5-(3-carboxymethoxyphenyl)-2-(4-sulfophenyl)-2H-tetrazolium) assay can be used to measure the change in cell viability caused by a change in metabolic activities. The cytotoxicity of free PEITC and/or CDDP, blank liposomes, drug-loaded liposomes toward human NSCLC cells (H596 and A549) and normal lung cells (WI-38 and BEAS-2B) (Figure 3 and Figure 4) were tested and compared using the MTS assay. All four cell lines were treated and incubated for 24 h. Figure 3 and Figure 4 show plots of the toxicity of various drugs, drug combinations and liposome formulations toward cells. The cytotoxicities of the various treatments were compared based on the average and standard deviation of each group (n = 6), which are shown as error bars in Figure 3 and Figure 4.

### 2.1. Effect of CDDP and PEITC Combination Therapy

In the cytotoxicity study using A549 cells (Figure 3A), the percent cell survival when treated with CDDP (5 µM) alone was 55.9 ± 3.4%, while treatment with PEITC (15 µM) alone resulted in a percent cell survival of 79.2 ± 3.8%. When treated with a combination of 5 µM CDDP and 15 µM PEITC (CDDP + PEITC), A549 cells had a percent survival of 46.2 ± 2.7%. The cytotoxicity associated with the combination of CDDP and PEITC was greater than those associated with CDDP or PEITC alone. As is shown in Figure 3B, the viability of H596 cells after treatment was similar to that of A549 cells. The percent survival after treatment with CDDP (5 µM) alone was 74.6 ± 9.2%, and the percent survival after treatment with PEITC (15 µM) alone was 84.9 ± 8.9%. Cells treated with a combination of 5 µM CDDP and 15 µM PEITC had a percent survival of 55.0 ± 9.5%. There are significant differences among groups CDDP + PEITC, CDDP and PEITC, as determined using a one-way ANOVA in R, for both A549 (*p* = 1.1 × 10^−14^) and H596 cell lines (*p* = 1.55 × 10^−7^) [20]. A Dunnett’s post hoc test accounting for multiple comparisons shows that for both cancer cell lines, the percent survival for the cells treated with a combination of CDDP + PEITC is significantly lower than free drug CDDP and PEITC, respectively (Table 3). The significant difference between the percent survival of CDDP + PEITC treatment groups and free CDDP treatment groups in both A549 and H596 cells confirmed the reported sensitization role of PEITC [8].

### 2.2. Effect of Liposome

The blank liposomes appeared to have no significant effect on cell growth with the percent survival of 92.6 ± 3.7%, 90.7 ± 9.1%, 85.5 ± 19.9%, and 96.0 ± 14.2% for A549, H596, WI-38 and BEAS-2B, respectively. Liposomes containing CDDP (5 µM) or PEITC (15 µM) showed greater toxicities toward A549 than the free drugs. The percent cell survival after treatment with liposomes loaded with CDDP (Lipo-CDDP) was 43.4 ± 4.0%, and for liposomes with PEITC (Lipo-PEITC), the percent cell survival was 64.6 ± 4.2%. Similarly, in H596 cells, the percent survival of the Lipo-CDDP treatment group (5 µM, 37.5 ± 8.9%) was lower than that of the free CDDP group (5 µM, 74.6 ± 9.2%), while cell percent survival of the Lipo-PEITC treatment group (15 µM, 69.8 ± 9.8%) was also lower than free PEITC (15 µM, 84.9 ± 8.9%). A Welch two-sample t-test found that the liposomal formulation of CDDP (Lipo-CDDP) exhibits a significantly higher cytotoxic effect than free CDDP on both A549 (*p* = 2.09 × 10^−5^) and H596 (*p* = 1.75 × 10^−6^) cell lines (Table 4). Similarly, the liposomal formulation of PEITC (Lipo-PEITC) has a significantly higher cytotoxic effect than free PEITC on both A549 (*p* = 5.86 × 10^−6^) and H596 (*p* = 1.15 × 10^−3^) cell lines. Lipo-PEITC, Lipo-CDDP and Lipo-PEITC-CDDP groups in the two lung cancer lines showed significantly higher cytotoxicity effects than free PEITC, CDDP and CDDP + PEITC groups, respectively, which indicated that liposomes can be used as an effective drug delivery vehicle.

### 2.3. Effect of Lipo-PEITC-CDDP on Cancer Cell Lines

When treated with liposomes containing 5 µM of CDDP and 15 µM of PEITC (Lipo-PEITC-CDDP), the A549 cells had a percent survival of 33.3 ± 2.9%, which was greater than that of CDDP + PEITC, Lipo-CDDP and Lipo-PEITC. Like A549 cells, the lowest percent survival of H596 cells (28.6 ± 6.3%) occurred when treated by 5 µM of CDDP and 15 µM of PEITC loaded in liposomes (Lipo-PEITC-CDDP), which was lower than that of CDDP + PEITC, Lipo-CDDP and Lipo-PEITC. There is a significant difference among groups Lipo-PEITC-CDDP, CDDP + PEITC, Lipo-CDDP, and Lipo-PEITC as determined using a one-way ANOVA for both A549 (*p* = 1.26 × 10^−14^) and H596 cell lines (*p* = 5.58 × 10^−10^). A Dunnett’s post hoc test accounting for multiple comparisons shows that for both cancer cell lines, the percent survival of the cells treated with Lipo-PEITC-CDDP is significantly lower than the free drug combination (CDDP + PEITC), as well as liposomal formulations of either single drug (Lipo-CDDP and Lipo-PEITC) (Table 5). Loaded with CDDP and PEITC, Lipo-PEITC-CDDP has the advantages of enhanced CDDP efficacy and effective liposomal drug delivery and showed the highest cytotoxicity against the two NSCLC cell lines.

A Welch two-sample t-test found that there is a significant difference (*p* = 1.16 × 10^−14^) between no treatment group and Lipo-PEITC-CDDP group for the A549 cell line. The t-value capturing the effect size of the difference is 68.0. Similarly, for H596, a difference between the two groups was found with a *t*-value of 23.9 and a *p*-value of 4.22 × 10^−8^. On the other hand, the effect sizes of the difference on normal cell lines were much lower (WI-38: *t* = 4.23, *p* = 5.10 × 10^−3^; BEAS-2B: *t* = 4.72, *p* = 4.07 × 10^−3^) (Table 6). This result shows that the Lipo-PEITC-CDDP drug therapy leads to a more significant cytotoxicity toward cancer cell lines as compared with normal cell lines.

Because of our 3:1 PEITC:CDDP ratio, we were able to use less cisplatin in the Lipo-PEITC-CDDP against NSCLC cells than in our last formulation, which was 2:1 PEITC: CDDP [16]. We were able to achieve similar toxicity profiles using less CDDP and a higher concentration of PEITC. Cisplatin is a known nephrotoxic agent, so reducing its concentration to get a similar toxicity profile in NSCLC cells is advantageous. For the two normal lung cell lines (Figure 4A,B), while the Lipo-PEITC-CDDP caused the lowest survival percentages, 59.0 ± 15.7% and 71.5 ± 11.0% for WI-38 and BEAS-2B, respectively, the cytotoxic effect of Lipo-PEITC-CDDP was much greater in NSCLC cells. Therefore, the PEITC and CDDP combined liposomal therapy has a high therapeutic index.

## 3. Materials and Methods

### 3.1. Materials

1,2-distearoyl-sn-glycero-3-phosphocholine (DSPC) and l-α-phosphatidylglycerol (egg, chicken) (EPG) were obtained from Avanti Polar Lipids, Inc. (Alabaster, AL, USA). Chloroform, phenethyl isothiocyanate (PEITC), cisplatin (CDDP) and dialysis sacks (12,000 Da) were purchased from Sigma-Aldrich (St. Louis, MO, USA). The 1,2-benzenedithiol (BDT) was obtained from Thermo Fisher Scientific (Waltham, MA, USA). Platinum (1000 ± 3 µg/mL), lutetium (1000 µg/mL) and gold (1000 µg/mL) standards were purchased from High-Purity Standards (North Charleston, SC, USA). The normal lung fibroblast cell line WI-38, normal bronchial epithelial cell line BEAS-2B and NSCLC cell lines H596 and A549 were purchased from the American Type Culture Collection (Manassas, VA, USA). CellTiter 96^®^ AQueous One Solution Cell Proliferation Assay (MTS) was from Promega (Madison, WI, USA).

### 3.2. Preparation and Characterization of Liposomes Encapsulated with CDDP and/or PEITC

First, 12.6 mg DSPC and 4.1 mg EPG were added to a round-bottom flask with 10, 16 or 20 µL of PEITC and 0.4 mL of chloroform. After 30 min of rotary evaporation, the dry, thin lipid film formed at the bottom of the flask was dissolved with 1 mL of normal saline solution containing 4, 6 or 8.33 µmol of CDDP. Then the mixture was sonicated for 2 min with a water-type sonicator (Branson Ultrasonics Corporation, Danbury, CT, USA) and vortexed for 30 s at 65 °C to obtain the resultant drug-encapsulated liposomes. Multilamellar liposomes were extruded 10 times through 200 nm and 100 nm polycarbonate membranes at 65 °C using an extruder (Avanti Polar Lipid, Inc., Alabaster, AL, USA) to achieve small unilamellar liposomes. The free CDDP and PEITC were removed using 50 kDa ultracentrifugation filter columns (Millipore Corporation, Billerica, MA, USA) at the speed of 3000 ×g for 20 min. A Zetasizer Nano (Malvern Instruments, Worcestershire, UK) was used to investigate the particle size distribution and zeta potential of the liposomes prepared using the dynamic light scattering technique. The structure and morphology of PEITC and CDDP co-encapsulated liposomes were characterized by Field Emission Scanning Electron Microscope (FESEM), Supra 55 VP (Zeiss, Oberkochen, Germany).

### 3.3. Determination of PEITC Loading in Liposomal-PEITC and Liposomal-PEITC-CDDP

The amount of PEITC loaded in the liposomes was determined using a 1,2-benzenedithiol (BDT) assay reported by Zhang with minor modifications [19]. To 1 mL of liposomal-PEITC suspended in potassium phosphate solution (100 mM, pH 8.5) or 1 mL of liposomal-PEITC-CDDP suspended in the same, was added 1 mL of 4 mM BDT solution in methanol. Then the 2 mL reaction solution was heated in a closed glass vial at 65 °C for 2 h, and its absorbance at 365 nm was measured using a UV-Vis spectrophotometer SpectraMax (Molecular Devices, Sunnyvale, CA, USA). A standard curve was established using PEITC (R^2^ = 0.98) in the same manner, and concentrations of PEITC in samples were determined based on the standard curve.

The PEITC drug loading was calculated using the equation:

(1) $$\mathrm{PEITC}\;\mathrm{loading}\left(\%\right)=\frac{\mathrm{Weight}\;\mathrm{of}\;\mathrm{PEITC}\;\mathrm{in}\;\mathrm{liposomes}}{\mathrm{Total}\;\mathrm{weight}\;\mathrm{of}\;\mathrm{lipids}\;\mathrm{and}\;\mathrm{PEITC}}\times 100\%$$

### 3.4. Determination of CDDP Loading in Liposomal-CDDP and Liposomal-PEITC-CDDP

Cisplatin loading was determined using inductively-coupled plasma-mass spectrometry (ICP-MS, PerkinElmer 350D, Waltham, MA, USA). Liposomal-CDDP and liposomal-PEITC-CDDP were digested with 70% nitric acid and heated at 70 °C overnight to evaporate. Then, the sample was re-dissolved in 70% nitric acid and diluted using deionized water, and the final samples for the ICP-MS test contained 2% nitric acid. Lutetium (20 ppb) was used as an internal standard.

The CDDP drug loading was calculated using the equation:

(2) $$\mathrm{CDDP}\;\mathrm{loading}\left(\%\right)=\frac{\mathrm{Weight}\;\mathrm{of}\;\mathrm{CDDP}\;\mathrm{in}\;\mathrm{liposomes}}{\mathrm{Total}\;\mathrm{weight}\;\mathrm{of}\;\mathrm{lipids}\;\mathrm{and}\;\mathrm{CDDP}}\times 100\%.$$

### 3.5. In Vitro Drug Release Studies

Different amounts of PEITC or CDDP were used to prepare liposomal-PEITC or liposomal-CDDP. The formulation that achieved the highest loading (%) of PEITC is referred to as “Lipo-PEITC”, the highest loading (%) of liposomal-CDDP is referred to as “Lipo-CDDP” and the liposomal formulation which combined the highest loading (%) of both PEITC and CDDP is referred to as “Lipo-PEITC-CDDP”. These three liposomal formulations were tested for their in vitro drug release and cytotoxicity. Drug release was studied using a dialysis method. Dialysis sacks were rinsed thoroughly using deionized water for 30 min, then 1 mL of Lipo-PEITC, Lipo-CDDP or Lipo-PEITC-CDDP was placed in the dialysis sack. The sack was fully immersed in a capped glass vial containing 20 mL of 1× PBS with 25% *v*/*v* methanol. A spin bar was added in the vial to achieve 100 rpm rotation speed. The temperature was set at 37 °C, and aliquots (100 µL) of the release medium were withdrawn for analysis at different time points (up to 24 h) and replaced with fresh medium. The absorbance of PEITC released from Lipo-PEITC and Lipo-PEITC-CDDP was measured using the same BDT assay as described above. The amount of CDDP released from Lipo-CDDP and Lipo-PEITC-CDDP was determined using ICP-MS.

The percentage of release of CDDP or PEITC at different time points was calculated using the equation:

(3) $$\mathrm{Release}\left(\%\right)=\frac{\mathrm{Cumulative}\;\mathrm{amount}\;\mathrm{released}}{\mathrm{Total}\;\mathrm{amount}\;\mathrm{in}\;\mathrm{liposomes}}\times 100\%$$

### 3.6. Cytotoxicity Studies

All cell studies were carried out in a humidified 37 °C, 5% CO_2_ (standard conditions) atmosphere incubator. For the two normal cell lines, the culture medium used for the WI-38 cells was the minimum essential medium (MEM) containing 100 µg/mL streptomycin, 10% fetal bovine serum (FBS), 2.0 mM l-glutamine and 100 IU/mL penicillin; the culture medium used for the BEAS-2B cells was bronchial epithelial basal medium (BEBM) with 10% FBS and supplements. The culture medium used for the two cancer cell lines, H596 and A549 cells, was Roswell Park Memorial Institute (RPMI) medium containing 100 µg/mL streptomycin, 100 IU/mL penicillin, 10% FBS, and 2.0 mM l-glutamine.

Nine groups (n = 6) were included to test the cytotoxicity toward each cell line. Two control groups were included: group 1, control group with only medium; group 2, control group with non-treated cells. Group 3–9 were cells with treatment: group 3, free CDDP; group 4, free PEITC; group 5, CDDP and PEITC (CDDP + PEITC); group 6, blank liposomes; group 7, Lipo-CDDP; group 8, Lipo-PEITC; group 9, Lipo-PEITC-CDDP. The cells were seeded at 5 × 10^3^ cells/mL (100 µL/well) in 96-well plates and allowed to grow for 24 h; after that, the medium was removed and replaced with 100 µL of medium containing the treatment suspensions, which was removed after an exposure time of 24 h and replaced with 100 µL of fresh medium. To each well, 20 µL of MTS solution was added and incubated for 2 h, and the UV-Vis absorbance was read at 490 nm. The percent survival of cells treated was calculated using the following equation:

(4) $$\mathrm{Survival}\left(\%\right)=\frac{A_{t}-A_{m}}{A_{c}-A_{m}}\times 100\%$$

where A_t_ is the absorbance of cells in treatment groups, A_m_ is the absorbance of medium alone and A_c_ is the absorbance of cells without treatment. All *p* values were calculated using Microsoft Excel *t*-test function (Redmond, WA, USA).

### 3.7. Statistical Analyses

All statistical analyses were conducted using *R* [20]. For the comparison of two group means, two-sample Welch’s *t*-tests were conducted using the t.test() function. The Levene test was carried out to test for homogeneity of variances using leveneTest() from the *car* package [21]. For comparison of more than two group means, one-way ANOVAs were conducted using the aov() function with a post-hoc Dunnett test carried out using the glht() function from the *multcomp* package [22].

## 4. Conclusions

Cisplatin is commonly used against NSCLC in the clinic; however, the negative side-effects associated with this metal-based drug urgently need to be addressed. We encapsulated CDDP and PEITC in liposomes, as this strategy has been shown to increase the circulation time of encapsulated CDDP and reduce its side-effects [17]. Furthermore, co-encapsulation of PEITC and CDDP in liposomes enhances their cytotoxicity toward NSCLC cells [16]. Release of PEITC and CDDP was studied and the cytotoxicity of this formulation against human NSCLC and normal lung cells determined. Lipo-PEITC-CDDP was significantly more toxic toward both NSCLC cell lines than toward the normal lung cell lines tested. Thus, we have prepared an efficacious therapy that is more toxic toward cancer cells than toward normal cells.

## Figures and Tables

**Table 1:** Percent drug loading and encapsulation efficiency (EE) of cisplatin (CDDP) and/or phenethyl isothiocyanate (PEITC) in liposomes.

DSPC (μmol)	EPG (μmol)	CDDP (μmol)	PEITC (μmol)	CDDP- Loading (%)	CDDP- EE (%)	PEITC- Loading (%)	PEITC- EE (%)
16	4	4	0	0.56 ± 0.29	60.5 ± 6.7	-	-
16	4	6	0	0.67 ± 0.33	79.8 ± 5.2	-	-
16 *	4	8.33	0	1.35 ± 0.27	83.9 ± 4.1	-	-
16	4	0	68	-	-	2.05 ± 0.21	26.7 ± 3.1
16	4	0	108.8	-	-	2.89 ± 0.32	31.5 ± 4.7
16 *	4	0	134	-	-	3.66 ± 0.35	37.0 ± 2.4
16 *	4	8.33	134	1.37 ± 0.18	84.3 ± 2.6	3.24 ± 0.47	34.7 ± 3.2

* Formulations that have the highest drug loadings, respectively, were chosen to be tested in cell studies.

**Table 2:** Size and zeta potentials of blank liposomes and liposomes loaded with cisplatin (CDDP) and/or phenethyl isothiocyanate (PEITC).

9 (μmol)	EPG (μmol)	CDDP (μmol)	PEITC (μmol)	Size (nm)	Polydispersity Index (PDI)	Zeta (mV)
16	4	0	0	116.3 ± 15.2	0.06	−41.0 ± 5.2
16	4	4	0	142.4 ± 26.4	0.08	−45.3 ± 7.4
16	4	6	0	149.2 ± 36.7	0.08	−46.3 ± 7.2
16 *	4	8.33	0	155.6 ± 29.5	0.09	−51.2 ± 6.5
16	4	0	68	139.2 ± 35.1	0.12	−50.6 ± 4.1
16	4	0	108.8	157.5 ± 28.3	0.13	−52.4 ± 7.8
16 *	4	0	134	165.6 ± 20.2	0.16	−54.5 ± 6.3
16 *	4	8.33	134	173.4 ± 26.8	0.22	−61.8 ±6.9

* Formulations that have the highest drug loadings, respectively, were chosen to be tested in cell studies.

**Table 3:** Effect of cisplatin (CDDP) and phenethyl isothiocyanate (PEITC) combination therapy.

Cell Line	Comparison	*p*-value *
A549	CDDP + PEITC vs. CDDP	2.4 × 10^−7^
	CDDP + PEITC vs. PEITC	<1 × 10^−10^
H596	CDDP + PEITC vs. CDDP	1.49 × 10^−5^
	CDDP + PEITC vs. PEITC	8.03 × 10^−8^

* Results of statistical analysis conducted using R (R Core Team, 2018).

**Table 4:** Effect of liposome.

Cell Line	Comparison	*p*-value
A549	Lipo-CDDP vs. CDDP	2.09 × 10^−5^
	Lipo-PEITC vs. PEITC	5.86 × 10^−6^
H596	Lipo-CDDP vs. CDDP	1.75 × 10^−6^
	Lipo-PEITC vs. PEITC	1.15 × 10^−3^

**Table 5:** Effect of liposomes containing both phenethyl isothiocyanate (PEITC) and cisplatin (CDDP) (Lipo-PEITC-CDDP) on cancer cell lines.

**A549**	
**Lipo-PEITC-CDDP vs.**	***p*-value**
CDDP + PEITC	<1 × 10^−6^
Lipo-CDDP	<1 × 10^−6^
Lipo-PEITC	<1 × 10^−6^
**H596**	
**Lipo-PEITC-CDDP vs.**	***p*-value**
CDDP + PEITC	<0.001
Lipo-CDDP	0.0367
Lipo-PEITC	<0.001

**Table 6:** Comparison of no treatment group with liposomes containing both phenethyl isothiocyanate (PEITC) and cisplatin (CDDP) (Lipo-PEITC-CDDP) on cancer and normal cell lines.

Cell Line	*p*-value	*t*-value
A549	1.16 × 10^−14^	68.0
H596	4.22 × 10^−8^	23.9
WI-38	5.10 × 10^−3^	4.23
BEAS-2B	4.07 × 10^−3^	4.72

## Abbreviations

NSCLC	non-small cell lung cancer
CDDP	cisplatin
PEITC	phenethyl isothiocyanate
DSPC	1,2-distearoyl-sn-glycero-3-phosphocholine
EPG	l-α-phosphatidylglycerol
